# Physicians’ roles in competency-based teaching: Do students recognize them?

**DOI:** 10.3205/zma001183

**Published:** 2018-08-15

**Authors:** Elvira Pippel, Elisabeth Narciß, Udo Obertacke, Renate Strohmer, Katrin Schüttpelz-Brauns

**Affiliations:** 1 Medical Faculty Mannheim, Heidelberg University, University Medicine Mannheim (UMM), Mannheim, Germany

**Keywords:** competency-based education, program evaluation, simulation training, undergraduate medical education

## Abstract

**Introduction: **Imparting the concept of physician’s roles with the help of new teaching formats is becoming increasingly important in medical education. The “ambulatory care simulation” was developed at the Medical Faculty Mannheim as a format to teach the roles of "medical expert," “communicator”, “health advocate”, “manager”, “team member” and “professional” in the practical year. During the “ambulatory care simulation”, students work through case scenarios with simulated patients focusing on the physician’s roles and subsequently discussing and reflecting on their experiences. Several measures are designated to ensure that each role is covered by the new teaching format. The present study investigates whether the physician’s roles are actually addressed during the “ambulatory care simulation” and whether the competency-based learning objectives are recognized by the students.

**Methods: **All participants in 12 of the 38 obligatory “ambulatory care simulations” signed informed consents to be filmed during the “ambulatory care simulation”. These videos were categorized using previously defined observation criteria. A total of 211 out of 224 students completed and handed in a one-minute paper at the end of the “ambulatory care simulation”. The answers to the question, “What have you learned?” have been assigned to competency-based learning objectives.

**Results: **Although instructors and students adhered to the guidelines in the recorded “ambulatory care simulations”, the most frequently addressed roles were “medical expert” and “communicator.” Two-thirds of the participants indicate learning outcomes that do not correspond to the previously defined learning objectives of the “ambulatory care simulation”.

**Discussion: **To ensure a thorough understanding and long-lasting appreciation of the physician’s roles, longitudinal integration of teaching interventions into the curriculum is to be favored over single teaching units. Instructors need intensive preparation for the unfamiliar construct of physician’s roles. The learning objectives must also be made more transparent.

**Conclusion:** Especially complex teaching formats need to be evaluated for success if they are to achieve their aims. Formative evaluations enable verification of whether the learning objectives are

1. addressed,
2. recognized and, finally,
3. achieved.

## Zusammenfassung

**Einleitung: **Die Vermittlung von ärztlichen Rollen mit Hilfe neuer Lehrformate gewinnt in der medizinischen Ausbildung immer mehr an Bedeutung. Das Lehrformat „Ambulanzsimulation“ wurde an der Medizinischen Fakultät Mannheim entwickelt, um die ärztlichen Rollen „Medizinischer Experte“, „Kommunikator“, „Gesundheitsfürsprecher“, „Manager“, „Teammitglied“, sowie „Professionell Handelnder“ im Praktischen Jahr zu vermitteln. In der „Ambulanzsimulation“ werden Fallszenarien mit Fokus auf die ärztlichen Rollen mit Simulationspatienten durchgeführt und im Anschluss besprochen und reflektiert. Verschiedene Maßnahmen sollten sicherstellen, dass die ärztlichen Rollen während der Lehrveranstaltung auch adressiert werden. Die vorliegende Studie soll untersuchen, ob die ärztlichen Rollen während der „Ambulanzsimulation“ tatsächlich adressiert werden und ob die kompetenzorientierten Lernziele von den Studierenden erkannt werden.

**Methode: **Von 12 der 38 verpflichtend durchgeführten Durchgängen der „Ambulanzsimulation“ liegen Einverständniserklärungen aller Teilnehmer für das Filmen der „Ambulanzsimulation“ vor. Diese Videos wurden anhand vorher definierter Beobachtungskriterien kategorisiert. 211 von 224 Studierenden haben im Anschluss an das Lehrformat ein One-Minute-Paper ausgefüllt und abgegeben. Die Antworten zu der Frage „Was haben Sie gelernt?“ wurde den kompetenzorientierten Lernzielen zugeordnet.

**Ergebnisse: **Obwohl sich in den aufgenommenen „Ambulanzsimulationen“ sowohl Dozenten als auch Studierende an die Vorgaben hielten, wurden v.a. die bekannten Rollen des „Medizinischen Experten“ und des „Kommunikators“ adressiert. Zwei Drittel der Teilnehmer geben Lernerfolge an, welche nicht den vorher festgelegten Lernzielen der „Ambulanzsimulation“ entsprechen.

**Diskussion: **Einmalige Lehrveranstaltungen zur Vermittlung ärztlicher Rollen bewirken keine nachhaltige Wahrnehmung der ärztlichen Rollen. Vielmehr müssen diese longitudinal in die Curricula integriert werden. Dozenten benötigen eine intensive Vorbereitung auf das ihnen unvertraute Konzept der ärztlichen Rollen. Die Lernziele müssen darüber hinaus transparenter gemacht werden.

**Schlussfolgerung: **Gerade bei aufwändigen Lehrformaten muss geprüft werden, ob sie ihr Ziel erreichen. Durch formative Evaluationen, kann überprüft werden, ob die Lernziele

1. adressiert,
2. wahrgenommen und letztendlich
3. erreicht werden.

## Introduction

There is an increasing demand for competency-based medical education both internationally and here in Germany. In the 1990s, the CanMEDS framework was developed in Canada to describe the different roles of a physician that are needed for the practical pursuit of the profession [1]. In 2014 the German Science Council called for training tailored to physician’s roles and competencies [2]. In 2015 the Medical Faculty Association passed the National Catalogue of Competency-based Learning Objectives for Undergraduate Medicine (NKLM) [http://www.nklm.de]. Here, the graduate profile is defined for the first time, thus delineating a core curriculum. This includes various physician’s roles that reflect the different expectations of future physicians (for example, in communicative and economic terms).

The simulation is a method to teach physician’s roles and prepare for contact with patients [3], [4], [5]. So far, simulation scenarios have been used in the context of practical skills, physical examinations and medical interviewing. Working with simulation patients (SP) is seen as meaningful and accepted by both students and practitioners [6].

Since 2006 at the Medical Faculty Mannheim of Heidelberg University, the final year is divided into quarters instead of the usual tertiary. In addition to the two mandatory rotations of internal medicine and surgery plus an optional rotation, the medical students complete one rotation in one of the four areas offered in ambulatory medicine, namely the fields of interventional surgery, conservative long-term care, oncology, or psychiatry & psychotherapy. This is intended to reflect the increasing focus on ambulatory care of medicine [7], [8]. The ambulatory quarter of the final year is carried out in specially selected ambulatory care clinics at the university hospital and its teaching hospitals, as well as at specialized centers and medical practices. The students are only deployed in ambulatory care clinics or practices where they can get to know patients over the course of their disease.

### “Ambulatory Care Simulation” as Teaching Format

As part of a MME project thesis, an “ambulatory care simulation” was developed to introduce medical students to the ambulatory care rotation. Designed as a competency-based teaching format, the aim of the simulation is to prepare students in the final year for their work in the ambulatory healthcare sector and, ultimately, for their future professional life [9]. In addition to the physician’s roles of “medical expert” and “communicator”, the final-year students in this class should also be made aware of their roles as “health advocate”, “manager”, “team member” and “professional”. The following learning objectives are defined:

Students are able to

1. … recognize life-threatening, avoidable conditions and apply strategies to avert them;
2. … integrate health promotion and prevention as basic elements of medical care;
3. … apply the principles of medical documentation in a manner appropriate to the situation;
4. … communicate appropriately with other health professions using medical terminology.

These general learning objectives are taken directly from the NKLM and are, as far as possible, addressed in each of the cases used in the “ambulatory care simulation”. Case-specific learning objectives, also taken from the NKLM, were derived from these learning objectives. Accordingly, the general learning objectives and case-specific learning objectives can be directly assigned to the physician’s roles. A list of examples of the general learning objectives, the case-specific learning objectives and the corresponding assignment to the physician’s roles in the NKLM can be found in Table 1 (Tab. 1).

The “ambulatory care simulation” was designed as a single mandatory course for all final year students in the ambulatory care rotation with four teaching units. Taking place between weeks 1 and 4 at the beginning of the ambulatory care rotation, the simulation was conducted in the skills lab (TheSiMa) of the Medical Faculty Mannheim. The students were divided into small groups (6-8 participants). To use resources most effectively, each SP played several cases per course (3 SP for 6 cases).

The following description refers to the revised version of the “ambulatory care simulation”. The original version can be found in Dusch et al. (2018) [9].

#### Carrying out the “Ambulatory Care Simulation”

In the simulations, two volunteer students from the small group were each asked to interview three unknown patients as a “physician” and to make diagnostic and/or therapeutic decisions in a given space of time (about 7 minutes per case). We discussed the cases and tasks required of the cases in detail with the individual case authors. Example versions of the cases for the students can be found in Table 2 (Tab. 2). Each student had 25 minutes to become familiar with the cases. In doing so, they were also able to search online for information needed to solve the cases. During the simulation, the “physicians” had access to a consultation room equipped with a desk, telephone, documentation system and a clock.

We included distractors in several cases to present an additional challenge. These were tasks that unexpectedly came up during the cases and either involved an additional, unexpected task, such as obtaining information over the telephone (e.g., previous findings from another hospital) or an actual disturbance, such as an uninvolved medical assistant coming in and asking a question in the middle of the case (played by one of the fellow students). When creating the cases, care was taken to ensure that the distractors were logically and practically integrated into the cases (see Table 3 (Tab. 3)).

The simulation was divided into two parts with three cases each (part 1: case 1-3, part 2: case 4-6). During these two parts, fellow students and instructors were required to use an observation sheet to evaluate various aspects of each case. In the subsequent debriefing, the sheets served as basis for the feedback from the instructors and fellow students, as well as for the content-related follow-up (example in Table 4 (Tab. 4)).

**Figure 1:**
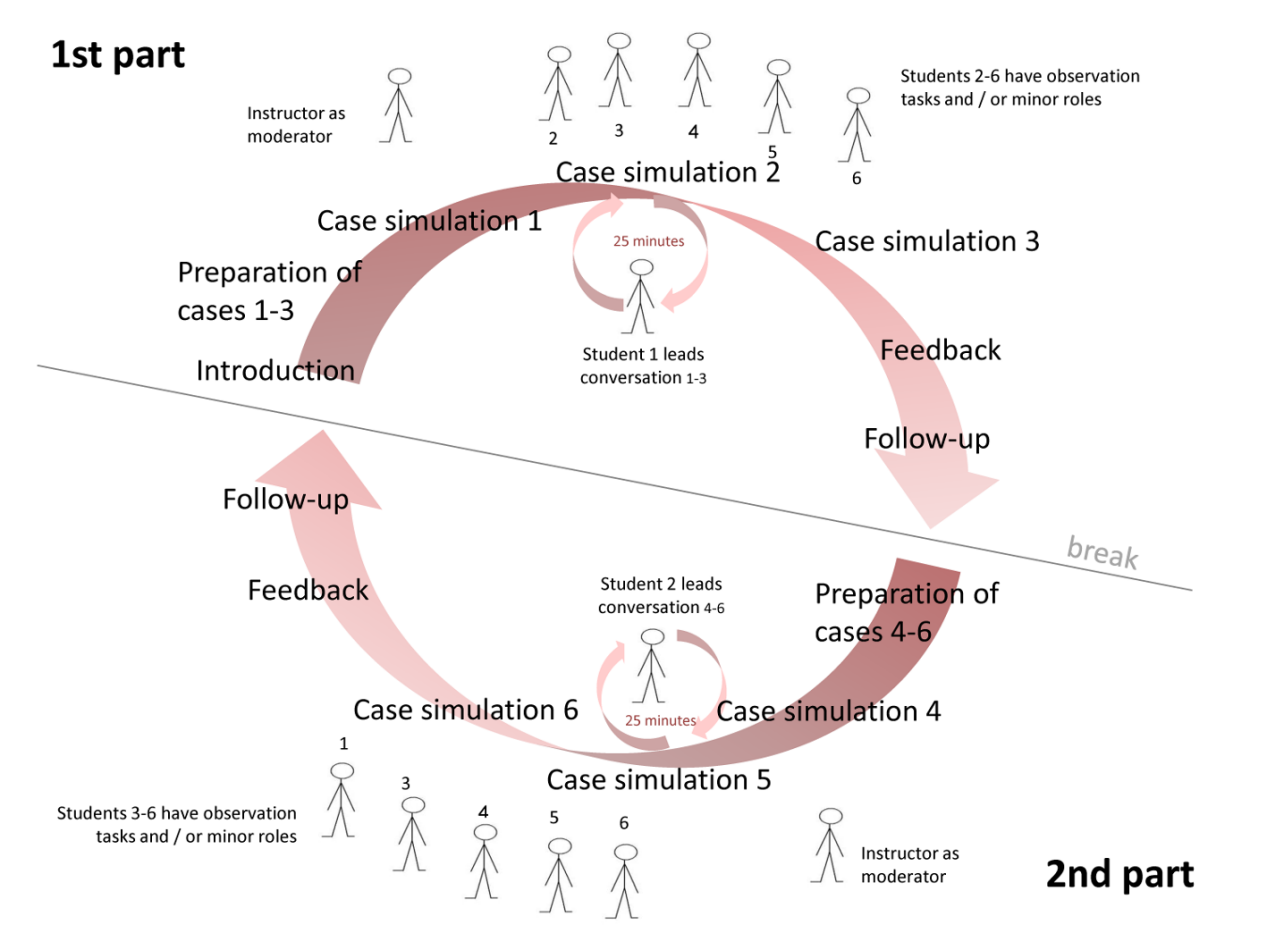
Schematic sequence of the ambulatory care simulation

The “physician” students received additional feedback from the SP [10]. Specialist content was summarized in sample solutions and handed out to all students following the ambulatory care simulation. An example of the workflow is presented in Figure 1 (Fig. 1). The instructor led the “ambulatory care simulation” by moderating the introduction and the feedback sessions; however, during the two simulation parts he remained a silent observer.

#### Preparation of the “Ambulatory Care Simulation”

Each instructor received an instructor's folder containing information on the introduction and a process overview of the “ambulatory care simulation” including precise descriptions of all cases and comments. Copies of all other materials were made available to both the students and the instructor. This included the tasks for the cases and the case-related observation sheets. To prepare for the “ambulatory care simulation”, each instructor was trained to precisely identify with the students the physician’s various roles in the cases. The preparations were adjusted to fit the schedule of each instructor and took up 45 to 60 minutes.

The students were familiarized with the “ambulatory care simulation” in the introductory phase at the beginning of the final year as well as during a presentation at the beginning of the ambulatory care rotation.

#### Addressing the Competencies in the “Ambulatory Care Simulation” as a Teaching Format 

Various measures were taken to address competencies regarding the physician’s roles using this teaching format. In the introduction, the instructors should

- explain all physician’s roles according to the NKLM.

In the preparation time for the cases the students should

- prepare the cases together in their small group (“team member”),
- search online for the case (“scholar”).

During the individual simulation runs of the “ambulatory care simulation”, time management should be practiced by:

- processing all three cases within 25 minutes (“manager”),
- proper handling of distractors (“manager”).

During the three cases in the “ambulatory care simulation”, the students and the instructor received one observation sheet per case. These contained

- observation tasks focusing on the addressed physician’s roles.

During the reflection after a case simulation

- feedback should be provided based on the observation sheets by the fellow students and by the instructor.

All these components should promote the competency-based focus of the “ambulatory care simulation” and thus the development of the physician’s roles.

#### How the “Ambulatory Care Simulation” functions as a Teaching Format 

Emphasis on competency-based teaching is steadily increasing in medical education. The question is whether these new teaching formats can actually promote competencies and represent different physician’s roles.

This study aimed to find out whether the “ambulatory care simulation” actually put the physician’s roles in the spotlight so that they were recognized by the students. This resulted in the following research questions:

- Are the physician’s roles addressed during the runs of the “ambulatory care simulation”?
- Are the competency-based learning objectives recognized by the students?

For the first question, the following points should be clarified:

- Does the instructor explain the physician’s roles in the introduction?
- Do the students prepare the cases as a team and via online research?
- Do “physician” students pay attention to the time (25 minutes per simulation part) and are the distractors used?
- Do fellow students and instructors use the observation sheets during the simulation parts?
- Does the instructor provide feedback based on the observation tasks?

## Method

### Sample

In 38 simulation runs, 12 different instructors with vast ambulatory care experience in different fields were involved (including pain ambulance, gynecology, urology).

From May 2014 to November 2015, a total of 224 undergraduate medical students attended the mandatory “ambulatory care simulation”. Four runs were performed according to the originally developed concept of “ambulatory care simulation” (MME project version), 34 runs according to the modified concept. This study refers to the revised “ambulatory care simulation”.

#### Material

For 12 runs of the “ambulatory care simulation”, instructors and all participating students signed an informed consent form to film the run. Videos were used to assess whether physician’s roles were addressed by the instructors during the introductions and debriefings and whether the intended physician’s roles were addressed in the cases. Seven different instructors moderated the runs. Two instructors had been involved with the teaching format from the beginning and also participated in the development of the cases. The other five instructors were introduced to the teaching format and moderated the runs at least two times.

A one-minute paper at the end of each run contained the open-ended question: “What did you learn?” and asked whether the previously defined learning objectives were recognized by the students.

#### Procedure

Each participant went through the “ambulatory care simulation” once at the beginning of his/her ambulatory care rotation. On several occasions students were informed about the possibility to voluntarily participate in the study. Signed consent forms were obtained from the participants after sharing the pertinent information. The simulations were filmed only if all participants had given their written consent. To allow a full view, both the group work and the simulation parts and the debriefings were recorded. After the run, students were asked to complete the one-minute paper. The Ethical Review Board of Medical Faculty Mannheim, Heidelberg University, approved the study (2014-554N-MA of 6.5.2014).

#### Statistical Analysis

The evaluation of the video recordings was based on a structured observation sheet developed by us covering the following categories:

Related to the run of the “ambulatory care simulation”

- Maintaining the structure of the “ambulatory care simulation” by instructors
- Introduction to the physician’s roles by instructors
- Use of the preparation time by students
- Maintaining time management during the simulation parts by the “physicians”
- Preparation of the cases in the small group
- Collection of information through online research

Related to the single cases

- Understanding the core issue of the case
- Completing the task regarding the physician’s roles
- Use of distractors
- Using the observation sheets during the simulation runs
- Feedback from the instructor on the main issues of the case
- Feedback from the instructor on the physician’s roles based on the observation sheet

One of the investigators analyzed the videos. In case of ambiguity, a second investigator could be included in the analysis in order to get a result.

The results of the video analysis were quantitatively evaluated.

Answers on the one-minute paper were categorized according to the four general learning objectives of the “ambulatory care simulation”. They were assigned to a learning objective when key terms of the respective physician’s role (NKLM) were mentioned, e.g. “documentation” which corresponds with the manager role. One person carried out the evaluation. Ambiguous answers were discussed with the head investigator.

## Results

### Descriptive Results

Table 5 (Tab. 5) shows that all investigators have adhered to the procedure protocol of the “ambulatory care simulation”.

The core issues of most cases were understood in all simulation runs. In case 4, however, these were only understood in one-third of the simulation runs (see Table 6 (Tab. 6)).

#### Definition of Competencies and Addressing Physician’s Roles in the Introduction by the Instructor

During the introduction, instructors failed to present the concept of competencies in all recorded scenario runs. In two videos instructors emphasized that “soft skills are trained here” without specifying them.

Furthermore, the instructors mentioned the physician’s roles of the “medical expert” and “communicator” most frequently during the introduction. The roles “manager” and “team member” were addressed in one-third of the “ambulatory care simulation” runs. The physician’s role of “health advocate” was addressed twice.

The detailed results are presented in Table 5 (Tab. 5).

#### Preparing the Cases by the Students in the Team and with Online Research

In all “ambulatory care simulation” runs, the students used the preparation time to work on the cases. They also searched for information online. Both the use of the preparation time and the online research were much more frequent in the first simulation part (cases 1-3) than in the second simulation part (cases 4-6). In some runs, the students organized themselves in tandems, so that two people each worked more intensively on one case. This was followed by a handover to the student who held the physician’s role in the subsequent simulation. Tandem collaboration was not intended at the beginning of the development, but was later taken up by the instructors. The detailed results are presented in Table 5 (Tab. 5).

#### Adhering to the 25-minute Time Limit per Simulation Part

Time management was maintained in about half of all simulation parts. In all other cases, students needed additional time. In simulation part 1 (case 1-3) the time was exceeded by three to eight minutes in 50% of the runs. In simulation part 2 (case 4-6) the time was exceeded by four to ten minutes in five cases.

The detailed results are presented in Table 5 (Tab. 5).

#### Use of the Distractors in the Cases

Except for case 4, the distractors were used in each case and each run.

The detailed results are presented in Table 6 (Tab. 6).

#### Use of Observation Sheets during Simulation Parts

In all simulation runs, the observation sheets were used by both students and instructors to take notes during the cases (see Table 6 (Tab. 6)).

#### Feedback Based on the Observation Tasks

The role-specific observation tasks were addressed in approximately one-third of all possible cases on the physician’s roles of “medical expert”, “health advocate”, and “manager”. The most frequent feedback was given on the physician’s role as “communicator”. The “professional” was mentioned in 42% of all possible cases in the feedback.

The detailed results are presented in Table 6 (Tab. 6).

#### Perception of Learning Objectives by Students

The evaluation of the student's one-minute paper, “What did you learn?” produced a wealth of different answers. Of the 211 submitted one-minute papers, a total of 224 responses were evaluated. Of these, only 77 answers could be assigned to the four learning objectives and 147 answers were categorized as “miscellaneous”. At least two-thirds of the students recognized other learning objectives than were intended in the “ambulatory care simulation“. Table 7 (Tab. 7) lists the matching of answers with the four general learning objectives.

Among the most widely recognized and primarily unintended learning objectives were time management (n=45) and interviewing (n=34). In addition, 17 students stated that they have gained a deeper insight into ambulatory medicine through the “ambulatory care simulation”.

## Discussion

Competency-based teaching is becoming increasingly important in medical education. However, it is usually not clear whether these new teaching formats actually are able to promote the physician’s roles. With the introduction of the “ambulatory care simulation” in the ambulatory care rotation during the final year at the Medical Faculty Mannheim, a complex competency-based teaching format going far beyond sole communication training with SP was piloted and scientifically evaluated. The aim of this study was to verify if the “ambulatory care simulation” addresses the different and intended physician’s roles during the simulation and whether the competency-based learning objectives are recognized by the students.

The video analysis showed that both the role of the “medical expert” and the role of the “communicator” are more frequently addressed by the instructors than the other physician’s roles. This may be due to the fact that these two roles are firmly anchored in the professional self-image of physicians, while the other physician’s roles are not yet so consciously recognized. In addition, there is a communication curriculum in Mannheim which is anchored longitudinally in the undergraduate training and for which the instructors are intensively trained.

An unintentional development was seen in the students’ teamwork during the preparatory phase in which there was separate case preparation with subsequent handover to the student in the physician’s role. We feel this behavior should be encouraged in future implementations, since it also addresses the physician’s role as “team member” and gives each of the three tandems a total of 25 minutes to prepare a case instead of the planned 25 minutes for a total of three cases. The collection of information through an online search was more frequent in the first simulation part than in the second simulation part. We cannot fully explain the decrease in motivation. Perhaps the case descriptions in the second simulation part contained too few concrete hints for targeted online searching, or the cases themselves were not good, or after the first simulation round the students felt that it was just another communication seminar in the final year. Since the students of the Medical Faculty Mannheim undergo a complete communication curriculum with SP during the clinical phase of their undergraduate training, their expectation might be to engage in “real patient contact” rather than SP.

Results show that the time management for the three cases per simulation part was a real challenge for the students. It turned out that students cannot accomplish such complex consultations within 7 minutes, especially those with built-in interferences. Experienced primary care physicians, who can easily handle simple consultations in 7 minutes (e.g., gastroenteritis / upper respiratory tract infection, dermatological/gynecological or surgical follow-ups), would take more than 10-15 minutes for the cases mentioned here. The aim should therefore be to extend the consultation period or simplify the cases. Since the issue of “time management” is not an explicit part of the curriculum, it should also be discussed in other courses.

The distractors were used in all cases except for case 4 (thoracic pain). As there were also problems with case 4 in other respects, this will need to be revised again. It is important to ensure that the technical complexity is minimized in favor of the competency-based approach.

Only a few students recognized the four general learning objectives. About two-thirds of the participants learned something different according to their own answers. The topics “time management” and “interviewing” were mentioned several times. Although these were not part of the learning objectives, they were addressed by the teaching format itself and should be included as explicit learning objectives.

All in all, we can conclude that the “ambulatory care simulation” addresses and promotes some of the physician’s roles; however, there must be a stronger focus on the physician’s roles, for both instructors and students. Complex technical content, as in the MDS case, must be reduced in order to present the physician’s roles more transparently. This could increase the learning success and make the teaching format more attractive for students since the relevance to the everyday life of the physician would be easier to understand.

This study has two limitations: participation in the “ambulatory care simulation” was mandatory, but the video recordings in this study were voluntary. All participants of one run (students and instructors) had to sign the informed consent to film the “ambulatory care simulation”. This was only the case in 12 of the 38 “ambulatory care simulation” runs. Biases due to voluntary participation can therefore not be excluded. In addition, the one-minute paper was evaluated by only one person.

The literature shows that the transfer of competencies must be longitudinal [10]. Single teaching units are not effective. By contrast, they have to be embedded in longitudinal curricula which explain the physician’s roles in the form of a learning spiral, establish a foundation of knowledge and skills, and then practice these skills in more complex settings. Furthermore, the instructors must be repeatedly sensitized in training sessions to the concept of competency-based instruction and the importance of the various physician’s roles.

The competency-based approach of the “ambulatory care simulation” as a teaching format was critically discussed. The simulation will be revised and embedded differently in the curriculum. The plan is to transfer it from the final year to the clinical phase of undergraduate education and to embed it longitudinally in the newly developed ambulatory care track, thus continuing to promote additional non-communicative and non-knowledge-related skills. This embedding aims at optimizing the effectiveness and usefulness of the teaching format [10], [11], [12], [13]. Instructor training will be revised. In addition to the organizational process and contents of the cases, the concept of competency-based education, the physician’s roles, and the role of the investigator as moderator and mediator in the “ambulatory care simulation” will be discussed in depth.

It is questionable whether other new teaching formats that are introduced would withstand such a critical evaluation. How we can find the balance between feasibility, complexity of cases, instructors’ everyday medical practice and necessary preparations for a new teaching format? Only when physician’s roles and the concept of competencies have been made clear to instructors and students alike can a possible transfer to medical practice be observed.

## Acknowledgements

We thank Dr. med. Martin Dusch who developed the original concept as part of his MME project thesis in collaboration with the Competence Center of the Final Year Baden-Württemberg and the Educational Research Team of the Medical Faculty Mannheim at Heidelberg University. 

Furthermore, we thank Dr. med. Magdalena Kowoll for the literature research and critical reading of the manuscript.

## Funding

The preparation and scientific evaluation of the “ambulatory care simulation” teaching format was funded by the Federal Ministry of Education and Research in the project entitled “MERLIN – Competency-based Teaching, Learning and Assessing in Medicine” (01PL12011L). 

## Competing interests

The authors declare that they have no competing interests. 

## Figures and Tables

**Table 1:**
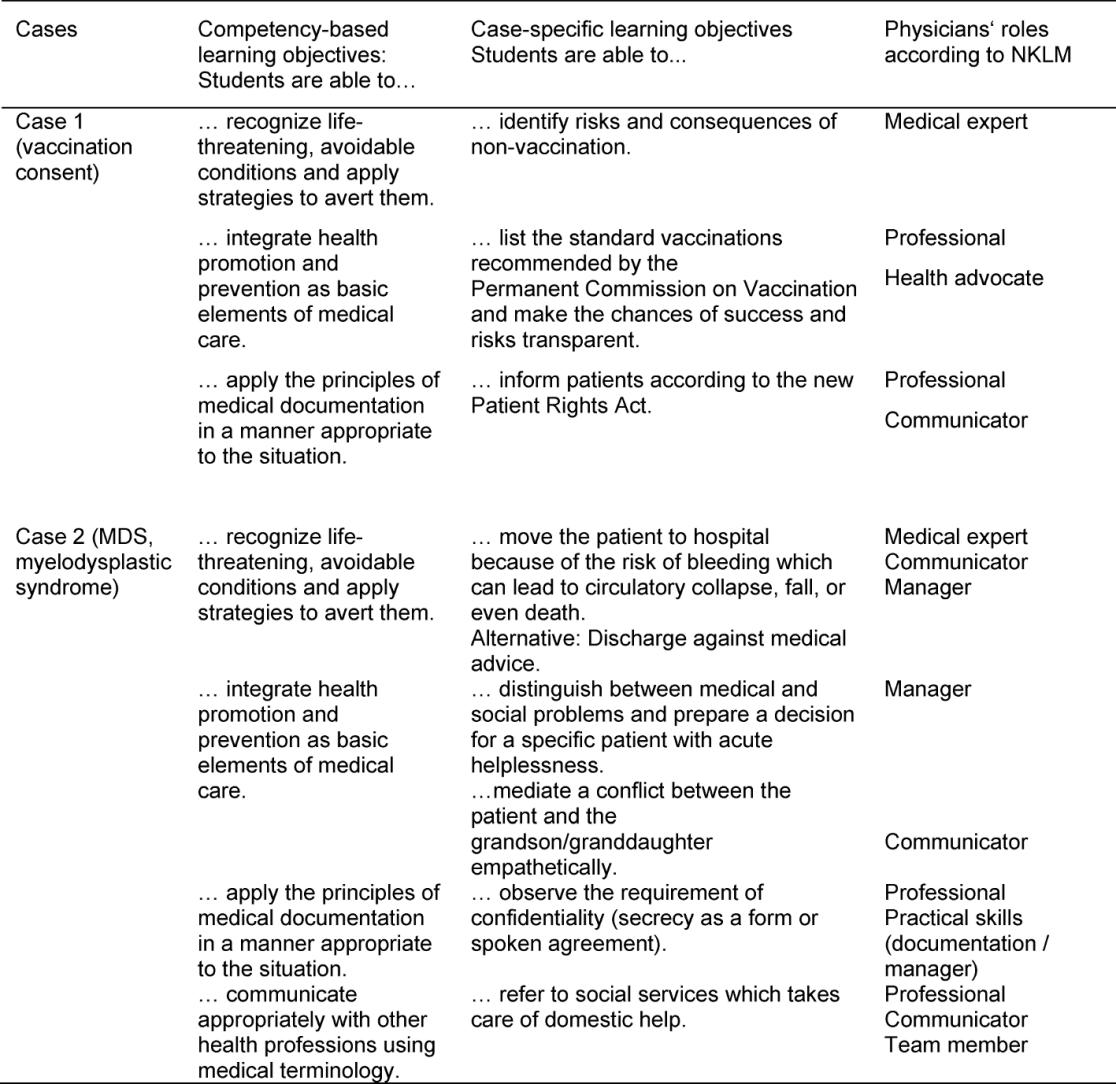
Examples of the learning objectives and roles according to NKLM (cases 1 and 2)

**Table 2:**
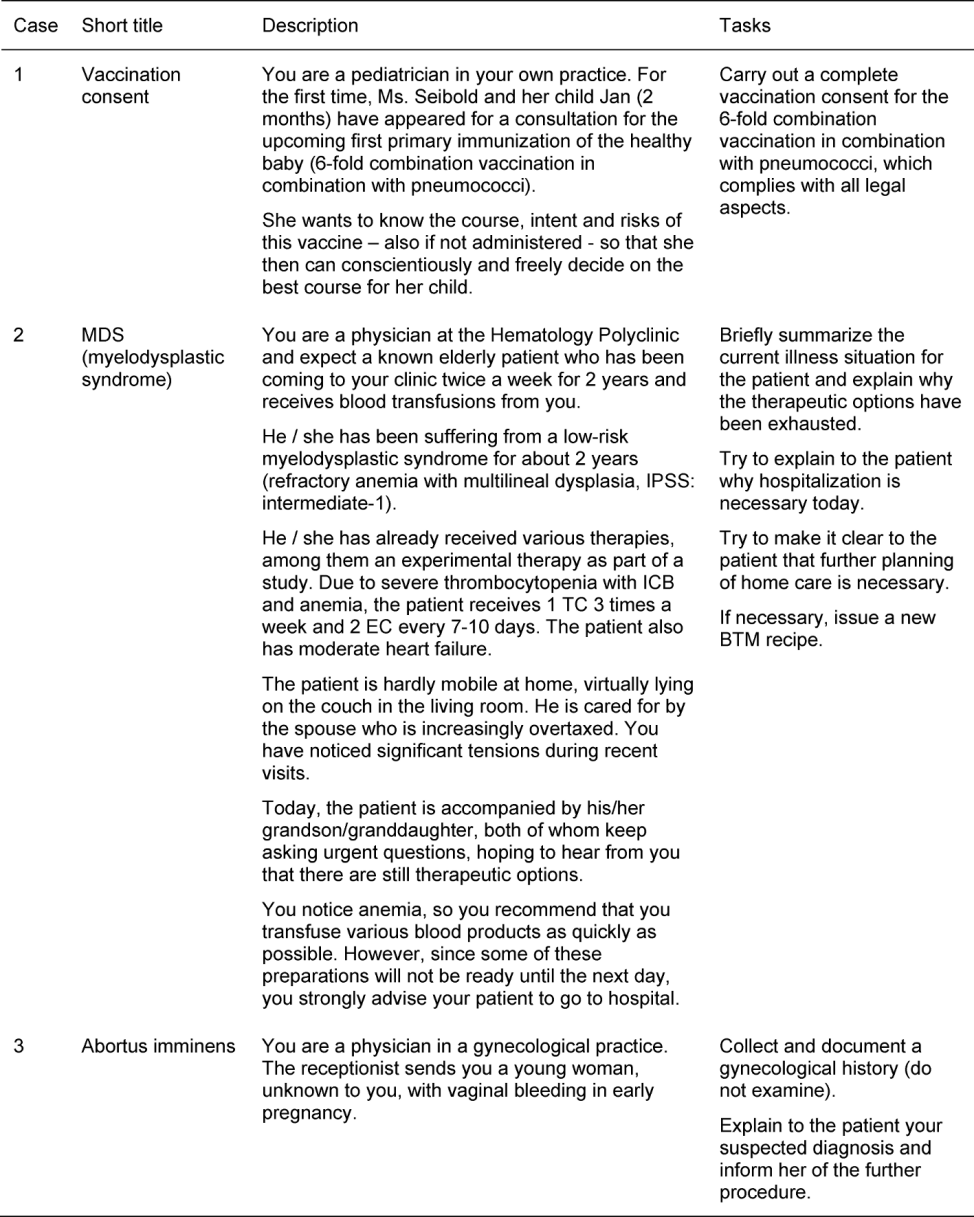
Exemplary cases and tasks for the students (case 1 to 3)

**Table 3:**
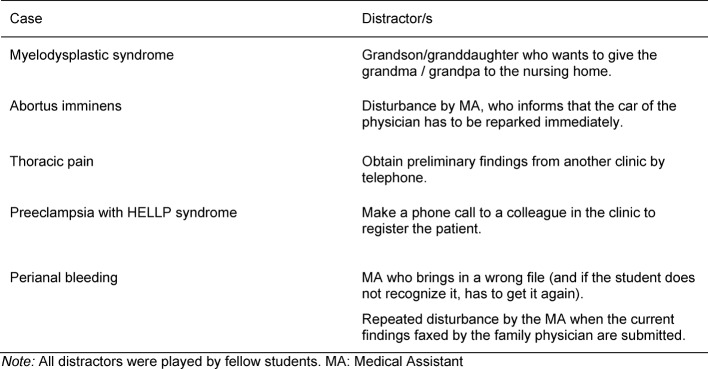
Overview of the distractors in each case

**Table 4:**
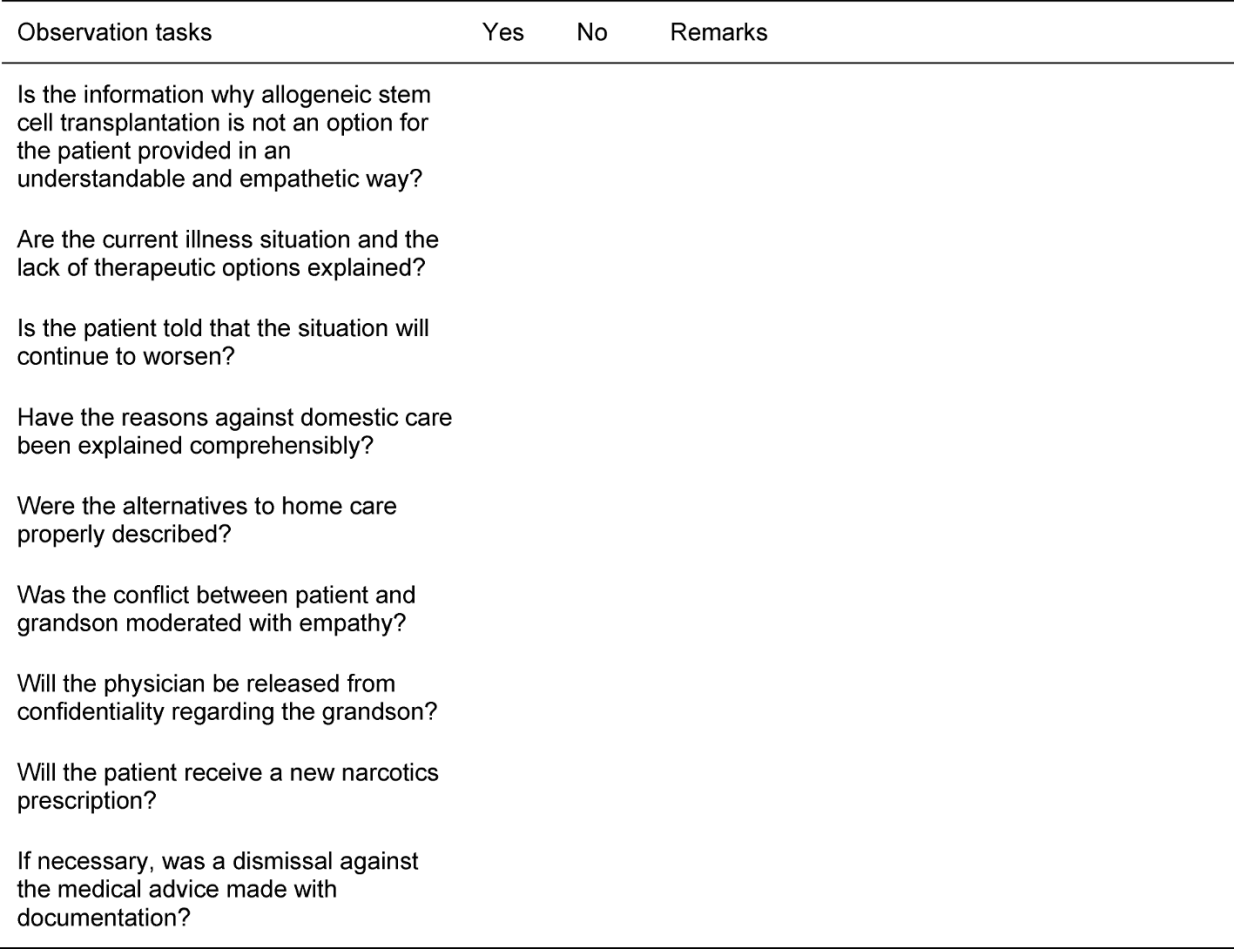
Example Observation tasks: Case 2 “Mrs./Mr. Maiwald” (MDS)

**Table 5:**
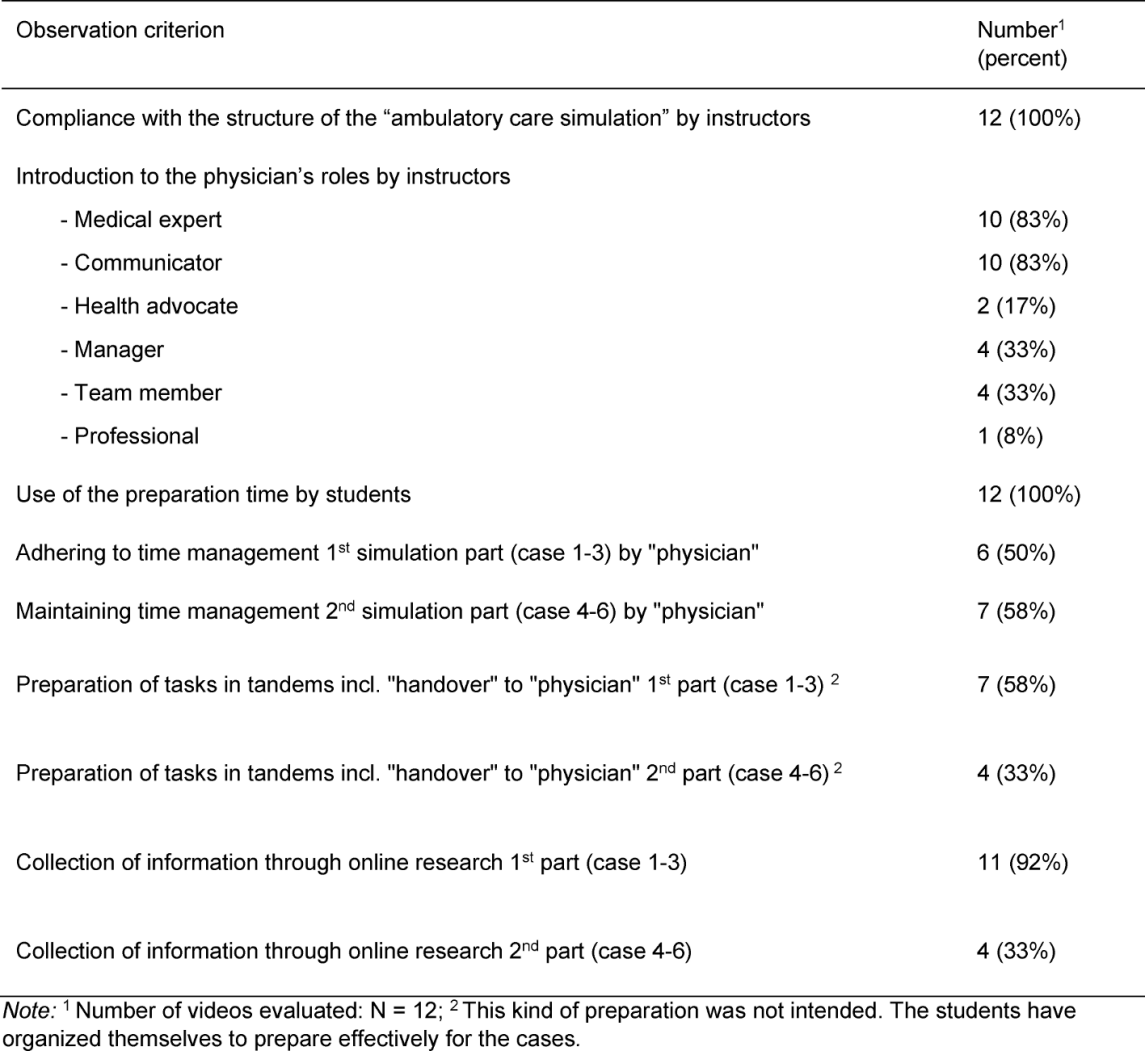
Results of the video analysis related to the “ambulatory care simulation”

**Table 6:**
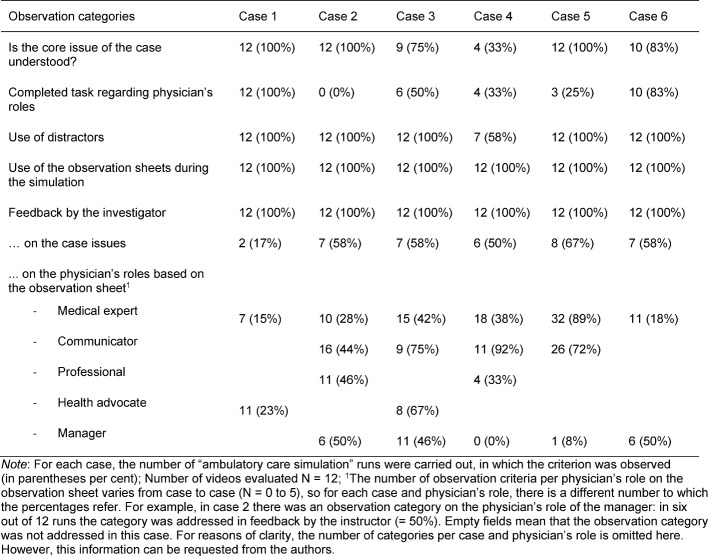
Results of video analyses regarding the single cases

**Table 7:**
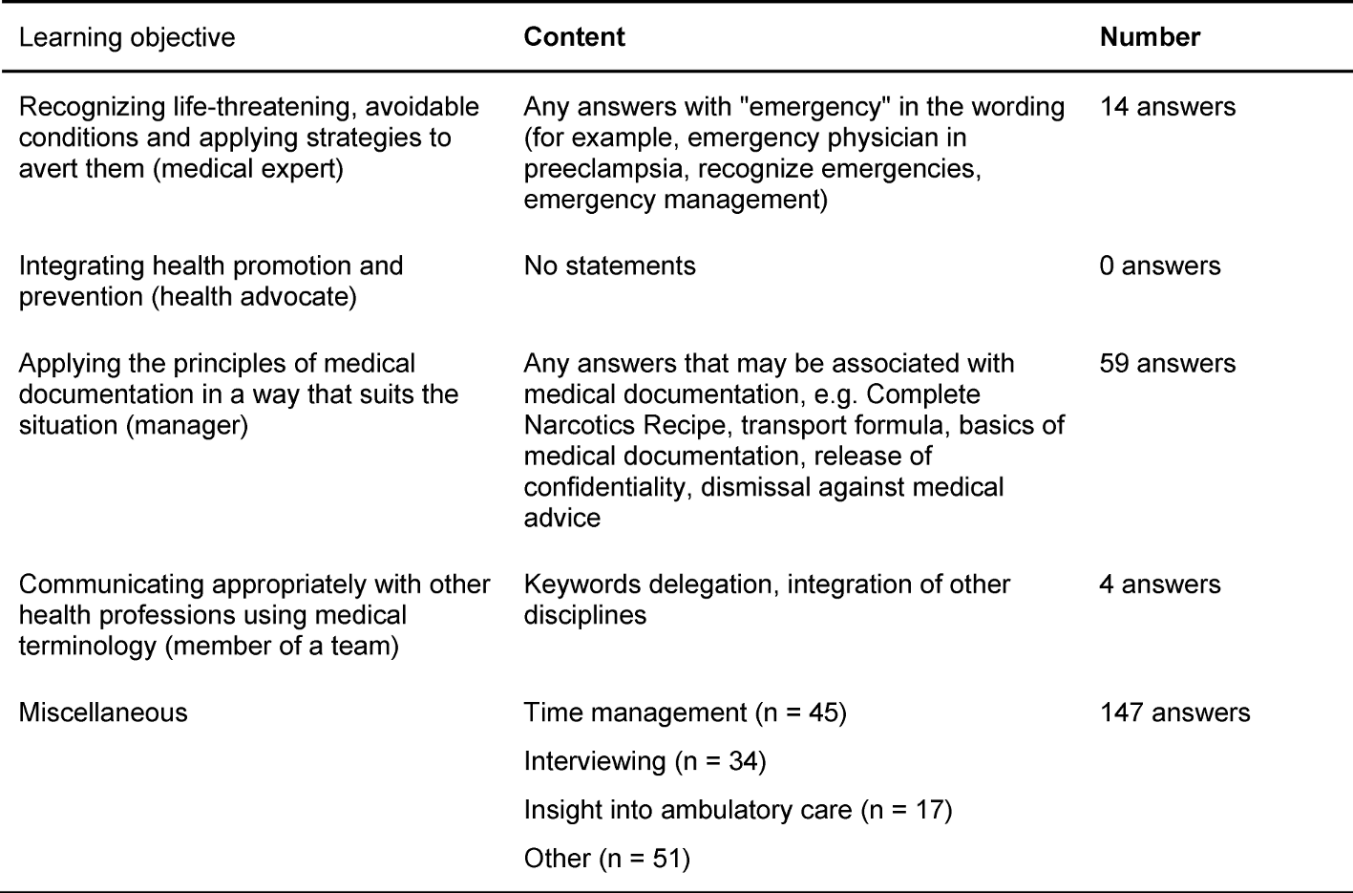
Assignment of the answers to “What did you learn?” to the four general learning objectives of the “ambulatory care simulation” (N=224)
